# Multi-Sensor Measurement of Cylindrical Illuminance

**DOI:** 10.3390/s26061991

**Published:** 2026-03-23

**Authors:** Michal Kozlok, Marek Balsky, Petr Zak

**Affiliations:** Department of Electrical Power Engineering, Faculty of Electrical Engineering, Czech Technical University, 166 27 Prague, Czech Republic; kozlomi1@fel.cvut.cz (M.K.); zakpetr@fel.cvut.cz (P.Z.)

**Keywords:** illuminance measurements, spatial illuminance characteristics, mean cylindrical illuminance, modelling factor, illuminance solid

## Abstract

**Highlights:**

**What are the main findings?**

- Approximation of cylindrical illuminance using multiple vertical detectors.
- Prototype of a multi-sensor device for cylindrical illuminance measurements.

**What are the implications of the main findings?**

- Enabling modelling-factor measurement with a single multi-sensor unit.
- Construction of a two-dimensional cross-section of the illuminance solid.

**Abstract:**

Spatial light field metrics, such as cylindrical illuminance, provide essential information for qualitative lighting evaluation, yet they remain far less common in practice than horizontal illuminance. To address this gap, we present a multi-sensor prototype that simultaneously measures horizontal illuminance *E_h_* and approximates mean cylindrical illuminance *E_z_* from a set of vertical illuminances uniformly distributed around a cylindrical surface. The device uses a flexible PCB wrapped around a support barrel, along with an inertial and magnetic measurement unit for orientation tracking. The measurements enable direct calculation of the modelling factor defined in the technical standard EN 12 464 and the visualization of the directional light distribution using polar plots and an illuminance solid. Results show that the prototype approximates mean cylindrical illuminance with high accuracy while preserving directional information, allowing the illuminance solid to be decomposed into vector and symmetric components. Compared with conventional approximation methods, the proposed multi-sensor approach reduces spatial error and yields richer data for lighting analysis. These findings indicate that multi-sensor systems can bridge the gap between theoretical spatial metrics and practical photometry and support the improved modelling evaluation and integration of qualitative lighting parameters into routine workflows.

## 1. Introduction

Research on the scalar and vector properties of the light field dates back to 1885, when Weber introduced luminance and illuminance distributions, along with early attempts to define average spherical illumination [1], now known as scalar illuminance [2]. Gershun’s 1936 work The Light Field [3] introduced the illuminance solid as a vector metric of qualitative properties of lighting as a fundamental description of the light field at a given point. Subsequently, hemi-scalar illuminance was formulated as a related scalar metric [4], followed by mean cylindrical illuminance in 1965 [5]. Lynes et al. extended Gershun’s paper in 1966 with the concept of the flow of light, which delivered the subject to a broader audience [6]. Cuttle [7] and Lynes [8,9] later developed the concept of the flow of light and multiple measurement methods of the flow of light were proposed [10], including Bunsen’s grease spot photometer [11,12].

In 1997, Cuttle proposed cubic illuminance [13] as a spatial distribution metric based on the Cartesian coordinate system, with its calculation and measurement methods. Parallel research by Prof. Habel at the Czech Technical University [14,15] explored cubic illuminance in 1993, although it was not published in English. Vector and symmetric components were explained by Cuttle [16,17] as the two major components of an illumination solid. Later studies by Cuttle [18], Straka [19], Mangkuto [20], and Xia et al. [21,22] demonstrated that cubic or multiple planar illuminances with varying levels of uncertainty can approximate cylindrical or scalar distributions. Mangkuto [23,24] and Xia et al. [25,26] compared multiple cubic to scalar approximation approaches across a large number of light field scenarios, with detailed error mapping and use-case proposals for specific approaches.

Despite extensive research, spatial metrics such as cubic, scalar, and cylindrical illuminance remain rarely used in routine photometry. Most technical standards prioritize horizontal illuminance for indoor workplaces and outdoor public areas, while spatial metrics, such as scalar or cylindrical illuminance, are usually mentioned only as recommendations [27,28,29,30]. The updated CEN standard EN 12 464 [31] highlights the mean cylindrical illuminance *E_z_* and the modelling factor, defined as the ratio of the mean cylindrical to the horizontal illuminance. Recommended modelling factor values for the indoor environment are from 0.3 to 0.6 [32], excluding cases where daylight contributes significantly. Values below 0.3 indicate a predominance of horizontal illuminance and can produce undesirable facial shadowing [33].

The cylindrical component *E_z_* represents the average vertical illumination critical for rendering three-dimensional features such as human faces, while the horizontal component (*E_h_*) represents the light falling on flat working surfaces. An optimal balance ensures that three-dimensional objects are adequately illuminated without being washed out by overly diffuse light or obscured by harsh overhead shadows.

This distinction is increasingly critical in the context of Human-Centric Lighting (HCL), which emphasizes the physiological and psychological impacts of illumination on building occupants [34]. While humans perceive and interact within a three-dimensional environment, evaluating a space based solely on a two-dimensional horizontal working plane is fundamentally insufficient. Incorporating spatial metrics like cylindrical illuminance provides a quantifiable measure of directional light flow. This ensures that the lighting design accurately supports human visual perception, enhances facial recognition for interpersonal communication, and improves overall spatial comfort [17,34].

At present, evaluating cylindrical illuminance in line with new technical standard recommendations [31] typically relies on calculations on a digital model of the lighting installation. While modern lighting design software can simulate these spatial metrics, digital models often rely on idealized surface reflectance and simplified, empty room geometries. In situ field measurements are therefore essential to account for real-world variables such as furniture occlusion, surface degradation, and dynamic daylight contributions, underscoring the need for practical spatial photometers.

Commercial photometers capable of measuring spatial distributions are rare. Cylindrical, semi-cylindrical, scalar, and semi-scalar photometer heads exist only as specialized accessories for portable light meters [35,36]. Devices for cubic illuminance *E*_06_ are not commercially available, and the measurement is either performed with six separate illuminance meters or by rotating a single receiver through the cube’s six directions [16,17,18,21,22].

However, the sequential rotation of a single receiver prevents simultaneous data acquisition, introducing temporal and alignment errors, while the alternative of using six separate meters demands equipment resources that are unrealistic for standard photometric practice. Consequently, modelling factor measurements usually require two instruments: one for horizontal illuminance and another for cylindrical illuminance.

To address these requirements, an illuminance measurement prototype was developed to simultaneously measure both horizontal illuminance *E_h_* and the cylindrical illuminance approximation *E_z_*, computed as the mean value of 36 evenly distributed vertical illuminances. Furthermore, the device is designed to resolve the vector component’s direction and magnitude in the control plane and directly calculate the modelling indicator proposed in EN 12464-1 [31].

## 2. Scalar and Vector Approach to Cylindrical Illuminance

**Figure 1:**
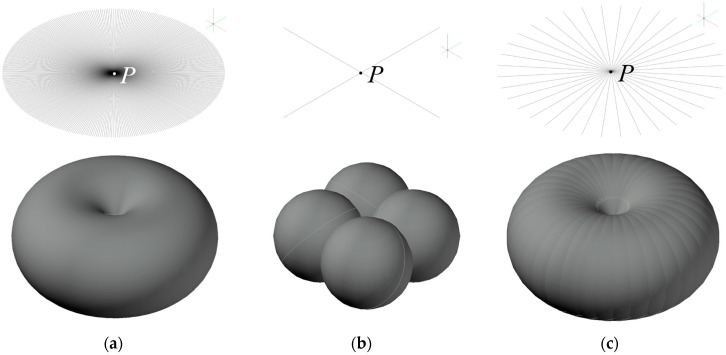
Graphical representation of (**a**) scalar cylindrical illuminance, (**b**) cylindrical illuminance *E*_04_ from 4 single detector illuminances, (**c**) cylindrical illuminance measured from 36 single detector illuminances. Above, the *E_N_* horizontal directions to the point *P*; below, the three-dimensional shape of spatial sensitivity is shown. The letter P denotes the central calculation point, and the colored axes indicate the spatial orientation (Red: X, Green: Y, Blue: Z cylindrical axis).

Scalar cylindrical illuminance in a real environment can be measured directly with a single detector with a specialized photometric head [35,36], as shown in Figure 1a. A close cylindrical illuminance approximation can be obtained by taking multiple measurements with a planar photometric head with a cosine corrector oriented to various directions around the vertical axis (Figure 1b), or by using a single head fitted with several detectors, as in Figure 1c. By analogy with the last case, scalar illuminance can be derived from repeated single-plane illuminance *E_n_* measurements taken from multiple directions at point *P*, or from axial rotation of a multi-sensor head.

The mean cylindrical illuminance *E_z_*, measured in lux (lx), at a given point *P*, is mathematically defined as the continuous integration of the light field’s luminance *L_ϑξ_* over the full spherical solid angle 4π sr. The incident light arrives through an elementary solid angle *dΩ_ϑξ_*, whose spatial direction is established by the elevation angle *ϑ* relative to the cylindrical axis *o* and the azimuth angle *ξ*. The sin *ϑ* multiplier serves as a geometric weighting function. Consequently, luminance originating from the horizontal plane (*ϑ* = 90°) yields the maximum contribution to the cylindrical evaluation surface, whereas luminance parallel to the vertical axis (*ϑ* = 0° to 180°) provides zero contribution. The resulting integral is subsequently normalized by a coefficient of 1/π to account for the cylindrical geometry (Equation (1)) [15].

(1) $$E_{z}=\frac{1}{\pi }\int_{0}^{4\pi }L_{\vartheta \xi }\cdot \sin\vartheta \cdot {d\Omega }_{\vartheta \xi }$$

**Figure 2:**
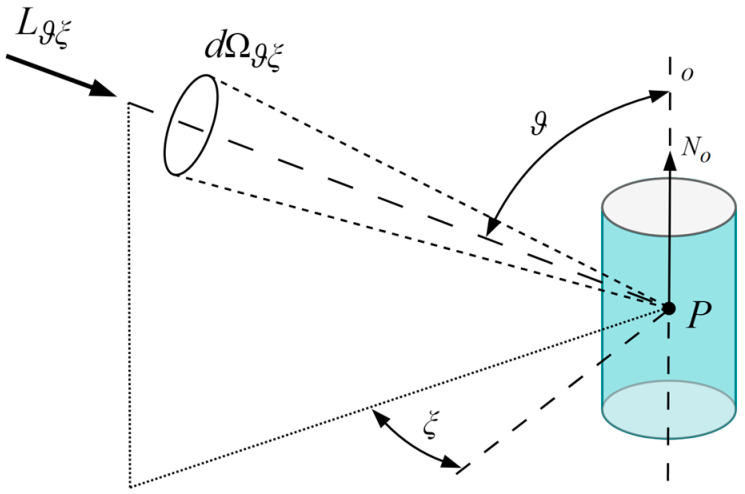
Geometrical parameters of cylindrical illuminance *E_z_* [15].

To visualize this mathematical formulation, the geometric parameters for the continuous evaluation of the light field relative to a vertical cylindrical surface are illustrated in Figure 2. The incident light arrives at the evaluation point *P* through an elementary solid angle *dΩ_ϑξ_*. The spatial direction of this incoming luminance *L_ϑξ_* is established by two angular coordinates: the elevation angle *ϑ* measured from the vertical axis *o* aligned with the normal vector *N_o_*, and the azimuth angle *ξ* within the horizontal plane.

The approximation of mean cylindrical illuminance *E_z_*′ [14] can be evaluated using *n* evenly distributed planar sensors around a vertical axis (Equation (2)). In this mathematical model, the incident light field at measurement point *P* is described by its luminance *L_ϑξ_* arriving through an elementary solid angle *dΩ_ϑξ_*. The incidence direction is defined by the elevation angle *ϑ* relative to the vertical axis *o*, and the azimuth angle *ξ_i_* relative to the normal vector *N_i_* of each respective planar sensor *I*.

(2) $$E_{z}\prime =\frac{1}{n}\sum_{i=1}^{n}\left[\int_{\Omega_{i}}L_{\vartheta \xi }\cdot \sin\vartheta \cdot \cos\xi_{i}\cdot {d\Omega }_{\vartheta \xi }\right]\;$$

**Figure 3:**
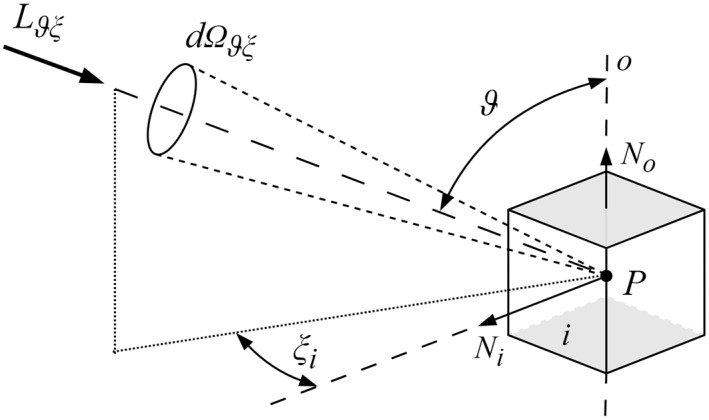
Geometrical parameters of cylindrical illuminance approximation—illuminance of four sides of a cube *E*_04_ [14].

In Equation (2), the integration domain *Ω_i_* represents the hemisphere visible to the *i*-th sensor, corresponding to incidence angles for which the cosine response is positive (cos *ξ_i_* ≥ 0). While the absolute magnitude of the measured illuminance depends on the elevation angle through the sin *ϑ* multiplier, the relative spatial error of the discrete approximation is dictated solely by the azimuthal cosine weighting (cos *ξ_i_*). Because true cylindrical illuminance evaluates the light field continuously over a perfectly circular cross-section, the discrete summation of *n* planar detectors inherently introduces directional sensitivity variations in the horizontal plane. To illustrate the mechanics of this geometric discretization, the simplest functional arrangement of the four-sensor configuration (*E*_04_) is visualized below in Figure 3. In this arrangement, the orthogonally positioned detectors approximate the cylindrical integration but generate significant spatial error. The top and bottom horizontal planes are treated as opaque surfaces that do not contribute to the discrete summation.

Since each planar detector’s output is weighted by the cosine of the angle of incidence, the combined spatial sensitivity yields a non-uniform, scalloped distribution. For any configuration of *n* sensors, the local minimums of the approximation occur in the normal directions of the detectors, defined for *k* = 0, 1, …, *n* − 1 in Equation (3).

(3) $$\xi_{min,k}=\frac{2\pi k}{n}$$

Conversely, the theoretical maximums occur in the diagonal directions exactly halfway between the sensors according to Equation (4).

(4) $$\xi_{max,k}=\frac{\pi (2k+1)}{n}$$

By evaluating the overlapping cosine responses at these extremes, the theoretical boundary for the true mean cylindrical illuminance *E_z_* can be generalized. This generalization requires an even number of sensors *n* ≥ 4, as a minimum of four planar sensors, aligned with the orthogonal axes (+*x*, −*x*, +*y*, −*y*) of the Cartesian coordinate system. The boundary inequality is given by Equation (5).

(5) $$\frac{n\sin\left(\frac{\pi }{n}\right)}{\pi }\;E_{0n}\leq \;E_{z}\leq \;\frac{n\tan\left(\frac{\pi }{n}\right)}{\pi }\;E_{0n}$$

**Figure 4:**
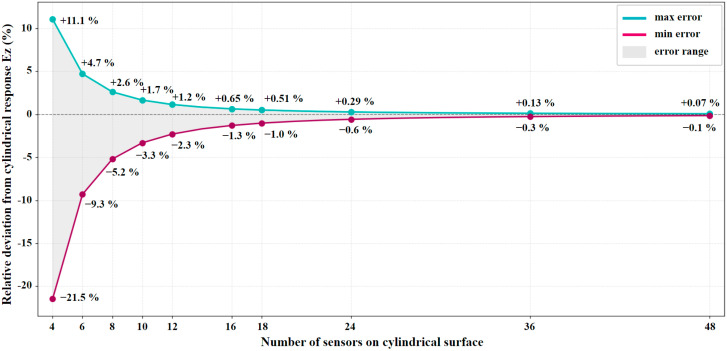
The dependence of spatial error on the number of sensors. Min Error is the error of illuminance approximation in the direction of the normal to the sensor, and Max Error is the error of illuminance approximation in the direction of the border between two sensors.

Applying this general rule to the four-sensor configuration yields the specific boundary limits for *E*_04_. As the number of sensors *n* increases, the trigonometric multipliers approach 1, significantly narrowing the spatial error. As illustrated in Figure 4, increasing the resolution to the proposed 36-sensor configuration reduces the theoretical spatial error limits to a maximum underestimation of −0.3% and an overestimation of +0.13%, bounding the maximum absolute spatial error to 0.3% [37].

Figure 4 shows the error as a function of sensor count *n* based on Equation (5). The process of selecting the number of sensors is also described in detail in [37]. The advantage of 36 sensors is a 10-degree angular measurement step.

Quality factors for a total characteristic of the illuminance meter performance are part of EN standard 13032-1 [38], yet spatial distribution characteristics deviation limits for cylindrical illuminance are not covered. German standard DIN 5032-7:2017 [39] specified class limits for illuminance meters, including scalar, cylindrical, semi-cylindrical, and semi-scalar illuminance spatial response for all four accuracy classes (L, A, B, C). The most accurate photometer class for in-field illuminance measurement (A) defines allowed cosine response deviation for standard illuminance < 1.5%, cylindrical illuminance < 5%, and scalar illuminance < 10% [39]. The spatial error of the prototype should therefore not significantly increase measurement uncertainties beyond the specified limits.

## 3. Multi-Sensor Measurement Device Prototype

**Figure 5:**
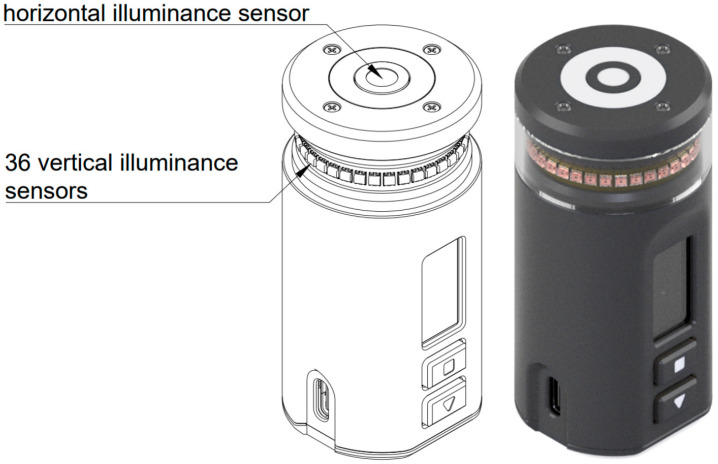
Proposed construction of a multi-sensor cylindrical illuminance sensor, drawing, and model render.

The properties mentioned above motivated the authors of this article to develop a single multi-sensor prototype that simultaneously measures horizontal illuminance *E_h_* and approximates the mean cylindrical illuminance *E_z_* by averaging the vertical illuminances measured by illuminance sensors uniformly distributed around a cylindrical surface (see Figure 5).

The device also estimates the magnitude and direction of the vector component and directly computes the modelling factor specified in EN 12464 [31].

**Figure 6:**
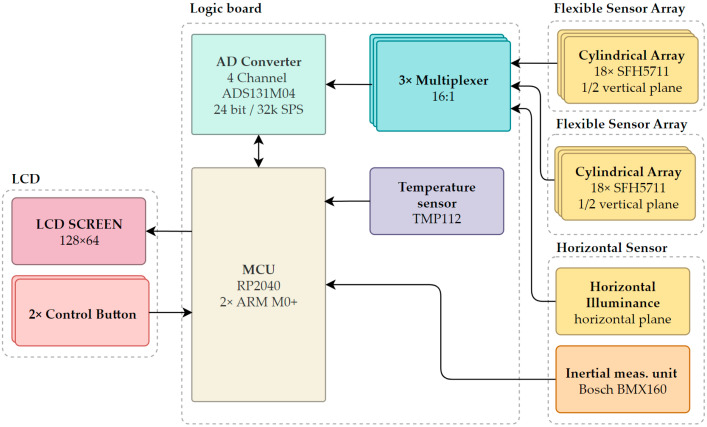
The topology of a cylindrical multi-sensor device prototype [37].

Compared to previous prototypes [37], the cylindrical sensor assembly comprises five PCB boards. Two flexible PCBs carrying the vertical sensors are wrapped around a cylindrical support barrel and connected to a main PCB that contains the main logic, power management, and data-acquisition stage, including ADCs and multiplexers. The horizontal illuminance sensor, status LED and IMMU are located on the top PCB. A user interface LCD and two buttons are situated on the horizontal PCB. The device supports battery-powered operation and USB data acquisition. The sensor topology follows Figure 6 [37].

**Figure 7:**
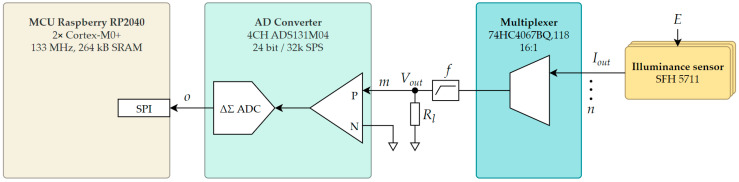
Signal flow through the multi-sensor system.

As illustrated in Figure 7, the Osram SFH 5711 sensor (ams OSRAM, Munchen, Germany) array signals *I_out_* are multiplexed, converted to voltage *V_out_* across a load resistor *R_l_*, and low-pass filtered (*f*) by the ADS131M04 (Texas Instruments, Dallas, TX, USA) AD converter (ADC). The number of ADC channels matches the number of multiplexers. The AD converter outputs are acquired by the MCU over the SPI bus as digital data frames.

**Figure 8:**
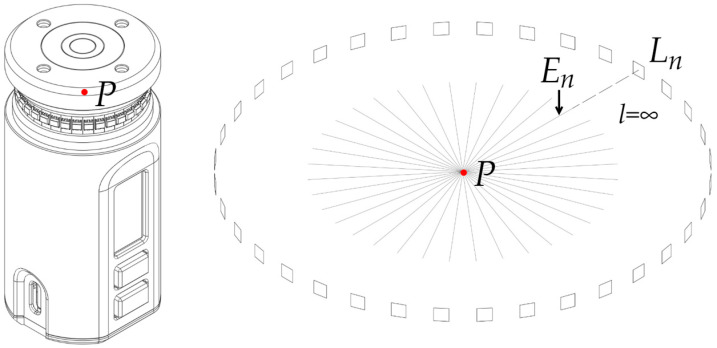
36 individual vertical illuminances on the cylindrical surface with a step of 10 degrees.

Real-time access to 36 individual vertical illuminances *E_n_* on the cylindrical surface at 10-degree steps (Figure 8) enables approximate evaluation of cylindrical illuminance evaluations consistent with the approaches of Gershun [3]. Semi-cylindrical illuminance and opposite illuminances can be obtained for any azimuth without moving the device.

An onboard inertial–magnetic measurement unit (IMMU) [40] provides approximate information about the horizontal plane level (spirit level) and heading of the device with respect to magnetic north (compass). Knowing the absolute device’s orientation offers the theoretical option of reconstructing the full illuminance solid by rotating the cylinder head about the horizontal axis.

After final assembly, the IMMU Bosch BMX160 (Bosch Sensortec, Reutlingen, Germany) is calibrated by leveling the device to trigger an internal FOC command for gyroscope bias, then rotating it through PJRC MotionCal software (21 May 2018 version) to compensate for housing interference using a 3 × 3 magnetometer mapping matrix. This yields a heading accuracy of ±1° to ±2.5° and a gyroscope offset of ±0.1°/s. The accuracy is expected to drift over time due to temperature fluctuations, sensor ageing or external field effects.

## 4. Results

The multi-sensor prototype is designed to simultaneously capture the horizontal illuminance *E_h_* and the mean cylindrical illuminance *E_z_* from a single instantaneous measurement. The horizontal component *E_h_* is primarily utilized for standard task area assessment according to EN 12464-1 [31], ensuring adequate light levels on the working plane.

**Figure 9:**
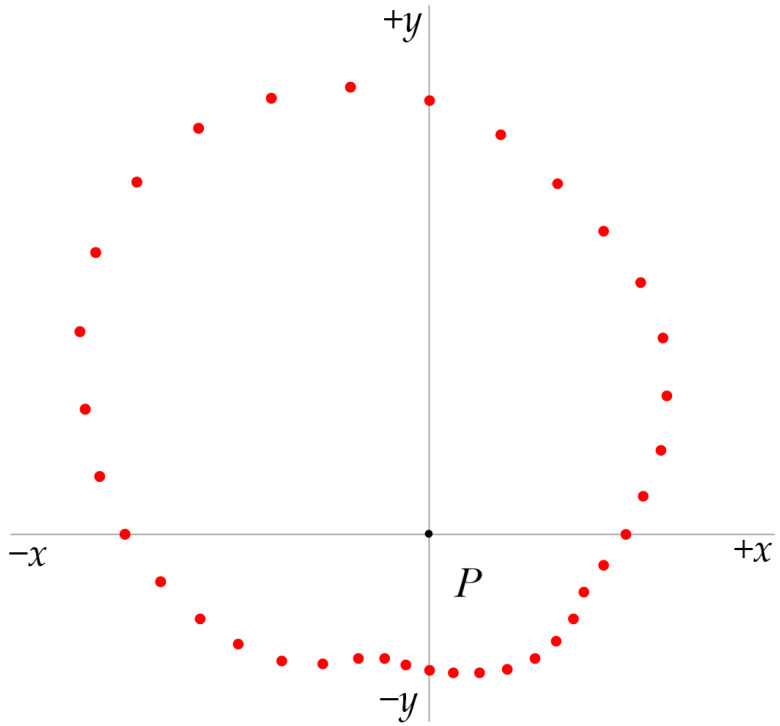
Polar chart of 36 single illuminance values measured on the horizontal plane by a cylindrical multi-sensor device.

Conversely, the 36 discrete vertical illuminance measurements serve a dual spatial purpose. First, their mean value provides the strictly scalar cylindrical illuminance *E_z_* for the direct computation of the modelling indicator (*E_z_*/*E_h_*) [31]. This indicator is firmly anchored in the EN 12464-1 standard, which recommends specific optimal ranges (typically 0.3 to 0.6) depending on the type of visual task to ensure three-dimensional rendering. Second, the full array of individual vertical measurements preserves the high-resolution directional data required to construct a two-dimensional cross-section of the illuminance solid (Figure 9).

To validate the prototype’s practical capability, a single-point example measurement was conducted under artificial indoor lighting conditions. The basic metrics of *E_h_*, *Ez*, and (*E_z_*/*E_h_*) are directly available on the integrated LCD screen, while the full *E_z_* vector dataset is accessible via a simple command protocol via the USB interface. The resulting quantitative data are summarized in Table 1.

**Figure 10:**
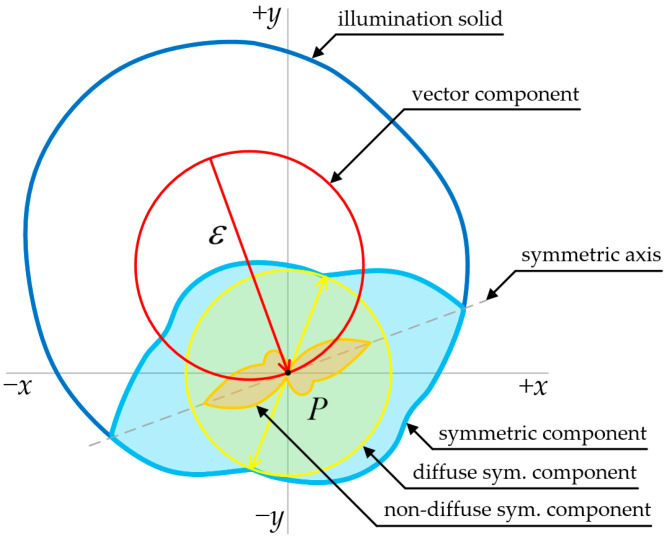
Two-dimensional illumination solid formed from 36 measured illuminance values.

Beyond a single cylindrical scalar metric of *E_z_*, this directional dataset allows for additional spatial visualization of the horizontal plane. The 36 single vertical illuminance *E_n_* values can be plotted in polar form (Figure 9) and interconnected to form a continuous outline representing a two-dimensional cross-section of the illuminance solid (Figure 10).

A concise description of the light field at a point *P* is given by a cross-section of the illuminance solid, constructed from illuminances *E_n_* measured in various directions (vectors) [3]. The illuminance solid is a three-dimensional surface whose radius in any direction equals the illuminance *E_n_* in that direction. Considering a two-dimensional section, the relevant illuminances lie in a single plane around point *P* (see Figure 10). Vertical illuminances sampled on a cylindrical surface with axis perpendicular to the section plane are measured by the multi-sensor cylindrical device prototype, and their mean value corresponds to the scalar value of mean cylindrical illuminance *E_z_*.

The illuminance solid cross-section (blue outer line in Figure 10) comprises two principal components: a vector component (red) and a symmetric component (light blue). The vector component’s magnitude and direction correspond to the illuminance vector $\vec{\varepsilon }$ (red arrow) [3]. The symmetric component can be further divided into its diffuse and non-diffuse symmetric components [16,17]. The two-dimensional illumination solid is considered on a horizontal plane, i.e., in all horizontal directions from point *P*. The vector component indicates the prevailing direction of illumination, whereas the diffuse component represents ambient light.

Table 1 presents the calculated modelling indicator (0.35), which quantifies the ratio of cylindrical to horizontal illuminance. However, it cannot express the directional distribution of the cylindrical component itself. This essential spatial context is provided by Figure 10. Visually, the ratio of the vector component (red) to the symmetric components (light blue) is well-proportioned, indicating that the measured *E_z_* (195.2 lx) consists of a balanced mix of direct lateral illumination and diffuse ambient light. Therefore, the multi-sensor prototype not only verifies that the scalar ratio is optimal for a visual task according to EN 12464-1 [31], but also visually confirms that the lateral light itself is free from extreme directional harshness.

While the visualization represents only a two-dimensional cross-section of the illuminance solid evaluated in a single horizontal plane, the absolute volumes of the individual components cannot be strictly quantified due to missing context from the full three-dimensional environment. Instead, this spatial visualization serves as a crucial qualitative diagnostic tool. The extracted vector component specifically provides a direct visual confirmation of the prevailing direction and relative strength of lateral illumination around a given point.

## 5. Discussion

The single-point measurement results presented in Section 4 practically demonstrate the feasibility of a multi-sensor approach for cylindrical illuminance measurements. By outputting both the scalar modelling indicator (Table 1) and the continuous two-dimensional illuminance solid (Figure 10), the prototype proves its capability to preserve crucial directional information.

This is consistent with previous theoretical frameworks by Gershun [3,4] and Lynes [2,8,9], which emphasize vector and symmetric components as fundamental descriptors of illumination. Although standards such as EN 12464 [31] recognize cylindrical illuminance for modelling purposes, its practical implementation has been limited by a lack of suitable instruments. The authors’ prototype addresses this gap by enabling simultaneous measurement of *E_h_* and *E_z_* and real-time computation of the modelling factor.

**Figure 11:**
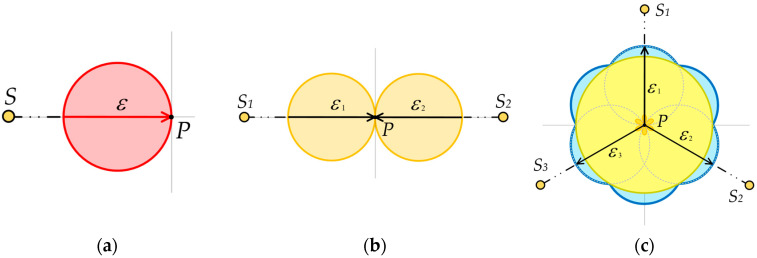
Simulations of outputs from a multi-sensor located at point P. (**a**) A single-point light source *S* resulting in an exclusive vector component (red). (**b**) Two opposite point light sources, *S*_1_ and *S*_2_, at a constant distance, resulting in an exclusive non-diffuse symmetric component (orange). (**c**) Three evenly spaced point light sources *S*_1_, *S*_2_ and *S*_3_ showing an inner diffuse component (yellow), grey dashed lines indicate response to each source, resulting in a solid cross-section (blue outline); all sources are considered at a constant distance with equal luminous intensity.

Simulation examples for a multi-sensor at point *P* illustrate typical cross-sections: an environment with a single point light source *S* (Figure 11a; dominant vector component), two opposite point light sources *S*_1_ and *S*_2_ (Figure 11b; non-diffusive symmetric component only), and three evenly spaced point light sources *S*_1_, *S*_2_ and *S*_3_ (Figure 11c; largely symmetric and diffusive component).

If light arrives uniformly from all horizontal directions, the two-dimensional cross-section of the illumination solid approaches a circle centered at the location of the multi-sensor (point *P*). In such an environment, objects cast no shadows, and distinguishing their surface structure (e.g., facial features) can be very difficult. If the multi-sensor is located in an environment with a dominant light source in one horizontal direction, the vector component (red in Figure 10 and Figure 11) of the illumination solid expands. A single horizontal point light source (Figure 11a) maximizes the modeling factor and produces sharp shadows. Conversely, with a single vertical point light source and negligible horizontal flow of light, the modeling factor approaches its minimum. Both single-point light source extremes (either vertical or horizontal) fall outside the recommended range of the modeling factor values.

The modelling ratio, as a quantitative indicator, is suggested to fall within the recommended range of 0.3–0.6. Values below 0.3 indicate excessive overhead lighting, while values above 0.6 indicate disproportionate lateral light (see Table 2).

While the device calculates the EN 12464 modelling factor as a scalar ratio, this number alone does not express the directional distribution of the cylindrical illuminance *E_z_*. This essential diagnostic context is provided by the polar plot, which visualizes the spatial components of *E_z_*. For a comprehensive lighting evaluation, the outputs should be interpreted concurrently, as demonstrated by the practical assessment in Section 4.

The polar plot serves as a qualitative indicator of how the vertical light is distributed. Even if a light field achieves an optimal modelling factor (e.g., 0.5), a polar plot dominated by a strong vector component (e.g., Figure 11a) indicates highly directional lateral illumination. Such a configuration produces harsh, asymmetric shadows on three-dimensional objects and facial contours. In contrast, the symmetric component within the plot confirms a balanced distribution of lateral light, which mitigates excessive contrast and creates an optimal visual environment for an observer to accurately perceive three-dimensional objects.

Compared with rotational or four-direction approximations, the 36-sensor configuration significantly reduces spatial error from more than 10% (4-sensor configuration) to less than 0.2%. These functions are relevant to workplace lighting, architectural design, and ergonomic assessment, where modelling quality influences visual comfort and perception. Future research should focus on metrological validation against reference-class photometers, algorithmic processing for automated vector decomposition, and sensor topology optimization for cost-effective production [41]. Integrating such devices into routine photometry could accelerate the adoption of spatial metrics and close the gap between standards requirements and practical lighting evaluation.

## 6. Conclusions

The research confirms that spatial illuminance metrics, particularly cylindrical illuminance, play a crucial role in qualitative lighting evaluation, yet technical limitations have hindered their practical implementation. The proposed multi-sensor prototype provides an accurate mean cylindrical illuminance approximation and directional light field analysis in a single measurement. This innovation aligns with normative modelling factor requirements and offers additional insights into illumination vector properties. To achieve broader adoption, further development is needed to improve measurement accuracy and reduce device complexity. Ultimately, integrating such solutions into standard lighting assessment practice can significantly enhance visual comfort and modelling quality in architectural and workplace environments.

## Figures and Tables

**Table 1:** Example measurement results in a single point.

Quantity	Value
Horizontal illuminance *E_h_*	556.3 lx
Cylindrical illuminance *E_z_* (as mean value of 36 vertical illuminances)	195.2 lx
Modelling indicator as ratio of *E_z_*/*E_h_*	0.35

**Table 2:** Modelling indicator assessment.

Modelling Indicator Value	Assessment of Appearance	Visual Effect
0 (min.)		exclusive overhead lighting, without lateral component
<0.3	Strong	strong overhead lighting, harsh shadows
0.3 to 0.6	Optimal	optimal balance of downward light for a visual task and lateral light for facial recognition
>0.6	Weak	shadows washed out, impression of flat space
1	Very weak to none	conditions of entirely diffuse environment
∞ (max.)		exclusive lateral light, without overhead component

## Data Availability

The data presented in this study are available on request from the corresponding author.

## References

[1] Weber L. (1885). Intensitätsmessungen des diffussen Tageslichtes. Ann. Phys..

[2] Lynes J.A. (1970). Cylindrical or scalar illumination?. Light. Res. Technol..

[3] Gershun A., Moon P., Timoschenko G. (1936). The Light Field.

[4] Gershun A. (1931). Characteristics of Conditions of Illumination. Trans. Opt. Inst..

[5] Epaneshnikov K.M., Sidorova T.N. (1965). The evaluation of light saturation of rooms of public buildings. Svetloteknika.

[6] Lynes J.A., Burt W., Jackson G., Cuttle C. (1966). The Flow of Light into Buildings. Light. Res. Technol..

[7] Cuttle C. (1971). Lighting patterns and the flow of light. Light. Res. Technol..

[8] Linnes J.A. (1975). Estimating scalar illuminance. Light. Res. Technol..

[9] Lynes J.A. (1975). The luminaire domain and the flow of light. Light. Res. Technol..

[10] Fuller M., Upton M., Whalen J. (1971). The photometry of the flow of light. Light. Res. Technol..

[11] Dale N., Broadbridge J., Crowther P. (1972). Measuring the direction of the flow of light. Light. Res. Technol..

[12] Lewis D.M. (1889). Bunsen’s Photometer. Nature.

[13] Cuttle C. (1997). Cubic illumination. Light. Res. Technol..

[14] Habel J., Straka T. Average Cubic Illumination. Proceedings of the 9th International Conference on Energy Effective and Ecologically Beneficial Lighting “Lighting ‘93”.

[15] Habel J., Dvořáček K., Dvořáček V., Žák P. (2013). Svetlo a Osvetlovani.

[16] Cuttle C. (2003). Lighting by Design.

[17] Cuttle C. (2015). Lighting Design: A Perception-Based Approach.

[18] Cuttle C. (2014). Research Note: A practical approach to cubic illuminance measurement. Light. Res. Technol..

[19] Straka T. (2001). Quality Assessment of Lighting Systems. Ph.D. Thesis.

[20] Mangkuto R. (2019). Uncertainty Analysis of Cylindrical Illuminance Approximation. Leukos.

[21] Xia L., Pont S., Heynderickx I. (2017). Light diffuseness metric Part 1: Theory. Light. Res. Technol..

[22] Xia L., Pont S., Heynderickx I. (2017). Light diffuseness metric, Part 2: Describing, measuring and visualising the light flow and diffuseness in three-dimensional spaces. Light. Res. Technol..

[23] Mangkuto R. (2019). A comparison of three approaches for determining scalar illuminance from cubic illuminance data. Light. Res. Technol..

[24] Mangkuto R. (2019). Research note: The accuracy of the mean spherical semi-cubic illuminance approach for determining scalar illuminance. Light. Res. Technol..

[25] Xia L., Xiao N., Liu X., Zhang T., Xu R., Li F. (2022). Determining scalar illuminance from cubic illuminance data—Part 1: Error tracing. Light. Res. Technol..

[26] Xia L., Gu Y., Liu X., Zhang T., Xu R. (2022). Determining scalar illuminance from cubic illuminance data. Part 2: Tests in real lighting environments and an approach to improve its accuracy. Light. Res. Technol..

[27] (2006). Interior and Workplace Lighting General Principles and Recommendations.

[28] (1992). Lighting for Buildings.

[29] (1997). Lighting for Sports Halls.

[30] (2024). Recommended Practice: Lighting Office Spaces.

[31] (2021). Light and Lighting: Lighting for Workplaces: Indoor Workplaces.

[32] Bean A. (1978). Modelling indicators for combined side and overhead lighting systems. Light. Res. Technol..

[33] Boyce P., Brandston H., Cuttle C. (2022). Indoor lighting standards and their role in lighting practice. Light. Res. Technol..

[34] Houser K.W., Esposito T. (2021). Human-Centric Lighting: Foundational Considerations and a Five-Step Design Process. Front. Neurol..

[35] PRC Krochmann. http://www.prc-krochmann.eu/Produkte/photometerkoepfe/.

[36] LMT Lichtmessetechnik Berlin. https://www.lmt.de/lmt-photometer-heads/.

[37] Kozlok M., Žák P. Photodetectors for cylindrical illuminance sensor. Proceedings of the 2020 21st International Scientific Conference on Electric Power Engineering (EPE).

[38] (2004). Light and Lighting: Measurement and Presentation of Photometric Data of Lamps and Luminaires—Part 1: Measurement and File Format.

[39] (2024). Deutsches Institut für Normung. Photometry—Part 7: Classification of Illuminance Meters and Luminance Meters.

[40] Zmitri M., Fourati H., Vuillerme N. (2019). Human Activities and Postures Recognition: From Inertial Measurements to Quaternion-Based Approaches. Sensors.

[41] Hrbac R., Kolar V., Novak T., Bartłomiejczyk M. Prototype of a low-cost luxmeter with wide measuring range designed for railway stations dynamic lighting systems. Proceedings of the 2014 15th International Scientific Conference on Electric Power Engineering (EPE).

